# Efficacy and Patient Tolerability Profiles of Probiotic Solution with Bisacodyl Versus Conventional Cleansing Solution for Bowel Preparation: A Prospective, Randomized, Controlled Trial

**DOI:** 10.3390/jcm9103286

**Published:** 2020-10-13

**Authors:** Youn I Choi, Jong-Joon Lee, Jun-Won Chung, Kyoung Oh Kim, Yoon Jae Kim, Jung Ho Kim, Dong Kyun Park, Kwang An Kwon

**Affiliations:** Gil Medical Center, Department of Gastroenterology, Gachon University College of Medicine, 405-760 1198 Guwol dong, Namdong-gu, Incheon 21565, Korea; cys7like@gilhospital.com (Y.I.C.); 7743@gilhospital.com (J.-J.L.); drgreen@gilhospital.com (J.-W.C.); kkoimge@gilhospital.com (K.O.K.); yoonmed@gilhospital.com (Y.J.K.); junghokimm@gilhospital.com (J.H.K.); pdk66@gilhospital.com (D.K.P.)

**Keywords:** screening colonoscopy, bowel preparation agent, probiotics

## Abstract

Although adequate bowel preparation is essential in screening colonoscopy, patient intolerability to bowel cleansing agents is problematic. Recently, a probiotic mixture solution with bisacodyl emerged to improve patient tolerability. We investigated the efficacy, safety, and patient tolerability profiles of probiotics with bisacodyl versus conventional polyethylene glycol (PEG) solution for bowel preparation for screening colonoscopies in healthy patients in this prospective, randomized, case-control study. In total, 385 volunteers were randomly assigned to receive 2 L of water + 200 mL of probiotic solution (case group, *n* = 195) or 4 L of PEG solution (control group, *n* = 190). The efficacy of the bowel cleansing was evaluated using the Ottawa scale system, polyp detection rate, and adenoma detection rate, and the patient tolerability profiles were assessed using a questionnaire. The demographics were not significantly different between groups. When the Ottawa score for each bowel segment was stratified into an adequate vs. inadequate level (Ottawa score ≤ 3 vs. >3), there were no statistical differences between groups in each segment of the colon. There were no significant differences in the polyp and adenoma detection rates between groups (38.42% vs. 32.42, *p* = 0.30; 25.79% vs. 18.97%, *p* = 0.11). The case group showed significantly fewer events than the control group, especially nausea, vomiting, and abdominal bloating events. Regarding the overall satisfaction grade, the case group reported significantly more “average” scores (95% vs. 44%, *p <* 0.001) and were more willing to use the same agents again (90.26% vs. 61.85%, *p < * 0.001). As patient compliance with bowel preparation agents is associated with an adequate level of bowel cleansing, a probiotic solution with bisacodyl might be a new bowel preparation candidate, especially in patients who show a poor compliance with conventional bowel preparation agents.

## 1. Introduction

Screening colonoscopy is associated with a reduced risk of colorectal cancer and, owing to the early detection of colon polyps and tumors, accounts for a 50–75% lower mortality rate [1,2,3,4,5,6]. According to recent studies, variation in the adenoma detection rate, a quality-controlled marker for screening colonoscopy, is closely associated with not only interval colorectal cancer risk but also the lifetime benefits and costs of colorectal cancer screening [2,7,8,9,10,11,12]. Although adequate screening colonoscopy quality is essential, 25–48% of colonoscopy cases do not achieve adequate bowel preparation [12,13,14,15,16,17,18,19,20].

In addition to patient co-morbidities and education levels for bowel preparation procedures, the patient tolerability profiles for bowel preparation agents are significantly associated with achieving adequate screening colonoscopy [13,14,16,21,22,23,24,25,26,27]. To improve patient preferences for bowel cleansing agents, several bowel preparation methods have been developed, such as a single dose vs. a split dose of purgatives; conventional large volume vs. ultra-low dose of bowel preparation agents; sulfate-free polyethylene glycol (PEG) vs. sodium phosphate solution; low-volume PEG plus ascorbic acid vs. high-volume PEG; and conventional PEG with bisacodyl, candy, coffee, or orange juice [28,29,30,31,32,33,34,35,36,37]. However, despite these attempts, there are still issues regarding the patient tolerability of bowel preparation agents, including taste, nausea, vomiting, and abdominal discomfort symptoms.

Recently, a probiotic mixture solution has emerged as a candidate for bowel cleansing agents. Probiotic mixtures are effective and beneficial in subjects suffering from evacuation disorders [38], and probiotic pretreatment as part of bowel preparation significantly improves the visualization of the colonic mucosa during colonoscopies in constipated patients [39,40].

Based on this, we used a prospective, randomized, controlled trial to compare the efficacy, safety, and tolerability profiles between probiotics with bisacodyl and a conventional 4-L PEG solution as bowel cleansing agents before screening colonoscopy.

## 2. Experimental Section

### 2.1. Institutional Ethics Review Board Approval of the Study Design

This study followed the tenets set forth in the Declaration of Helsinki. The protocol used in this study was reviewed and approved by the institutional review board of the ethics committees of Gil Medical Center (GMC) (IRB approval number of GMC: GBIRB2013-69). All the research was performed in accordance with national guidelines and regulations. This study has been registered with the Clinical Research Information Service (CRIS) (number: KCT0000954).

All the participants provided informed consent to voluntarily participate in the study with full knowledge of the relevant risks and benefits. All the study participants had all information that might reasonably influence their willingness to participate in a form that they could understand and comprehend.

### 2.2. Study Population

All the patients who attended the Health Promotion Center of Gachon University GMC were enrolled in the study. This was a single-blind, randomized, controlled case-control study using a probiotic solution (Lactosclean-Y^R^, Medical Optics, Seoul, Korea) with bisacodyl vs. a conventional PEG solution (Colyte-FR, Taejoon, Seoul, Korea).

The inclusion criteria were: (1) voluntary participation in this study with full knowledge of the relevant risks and benefits; (2) written informed consent; (3) aged over 20 and under 65 years old; and 4) no known medical history. Exclusion criteria were: (1) aged under 20 or over 65 years old; (2) a history of abdominal surgery, including gastric or bowel resection; (3) previously treated for electrolyte imbalance; (4) a history of any type of cancer, diabetes type I, abnormal liver function (liver cancer, liver cirrhosis, chronic liver disease, ascites, hepatic encephalopathy history, and esophageal/gastric varix history), or chronic inflammatory bowel disease or treatment owing to acute disease involving the bowel; (5) acute or chronic renal failure (glomerular filtration rate < 30 mL/min); (6) chronic heart failure (New York Heart Association Class > 2); (7) a history of cardiac disease (cardiac valvular disease, arrhythmia, myocardial infarction, unstable angina, and stable angina) or active or chronic pulmonary disease; and (8) taking any routine medication, including immunosuppressive drugs.

A coded envelope was used for the randomization of participants to receive either probiotic solution with bisacodyl (case group) or a conventional 4-L PEG solution (control group).

### 2.3. Endoscopist Profiles

Colonoscopies were performed by six expert endoscopists who had more than 10 years of experience in endoscopy and had experienced more than 20,000 colonoscopy cases. Endoscopists used a Pentax video colonoscope (EPK-i, EC-3490Fi, EC-3890Fi; Asahi Optical Co. Ltd., Tokyo, Japan). At the time of colonoscopy, the endoscopist was blinded to the preparation solution.

### 2.4. Bowel Cleansing Agent: Probiotic Solution with Bisacodyl vs. A Conventional 4-L PEG Solution

The participants who were allocated to the case group used a probiotic mixture solution (Lactosclean-Y^R^) with bisacodyl as a bowel preparation agent before the screening endoscopy. Lactosclean-Y^R^ is a solution composed of a concentration of *Bifidobacterium longum* with lactose, yogurt powder, oligosaccharides, powdered skim milk, Chinese pearl barley, citric acid, and vitamin C. The participants allocated to the case group were encouraged to take two tablets of bisacodyl (10 mg) at 7:00 a.m. on the day before the colonoscopy, followed by 200 mL of probiotic solution with 2 L of clear water to be consumed over 2 h from 8:00 p.m. Patients in both groups were advised to have a water gruel lunch on the day before the colonoscopy and fast until the colonoscopy. All the colonoscopies were performed in the morning.

The participants allocated to the control group consumed 4 L of PEG solution over 4 h from 6:00 p.m. on the day before the colonoscopy.

### 2.5. Outcome Variables: Efficacy, Safety, and Patient Tolerability Profiles

The efficacy profiles of the bowel preparation agents were evaluated using the bowel cleansing quality, polyp detection rate, and adenoma detection rate. The quality of bowel cleansing was determined using the Ottawa scale system [41]. The Ottawa scale was constructed with the degree of cleaning segment of the colon (left colon, transverse colon, and right colon) and the amount of fluid in the entire colon [41]. The score was “excellent, 0 points” when only a small amount of clear fluid remained in the colon; “good, 1 point” when some clear fluid remained but a good view of the mucosa was achievable; “fair, 2 points” when a small amount of semifluid stool was present that could be removed by blow liquid, rinsing, or suction; “poor, 3 points” when a significant amount of semisolid stool was present that could not be removed by rinsing or suction; and “inadequate, 4 points” when a semisolid or solid stool led to an incomplete colonoscopy [41]. The amount of liquid in the entire colon was assessed from 0 to 2 points: low (0 points), moderate (1 point), and high (2 points) [41]. In addition to the Ottawa bowel preparation scale, the polyp and adenoma detection rates were used to evaluate the efficacy of the bowel cleansing agents [11,42,43,44].

The safety profiles of the bowel cleansing agents were evaluated by recording uncomfortable symptoms from participants who completed bowel preparation and vital sign monitoring. Before the colonoscopy, all the participants who completed bowel preparation were asked to complete a questionnaire on any adverse events experienced owing to the bowel preparation agents. The questionnaire included closed- and open-ended questions on the symptoms during bowel preparation. Closed-ended questions asked about symptoms of (1) nausea/vomiting, (2) abdominal discomfort/pain, (3) abdominal bloating, (4) headache, and (5) dizziness, with ratings on a scale of 0 to 3: “0: never or rarely”, “1: mild”, “2: moderate”, and “3: severe”. Vital signs were measured before (30 min before), during, and after (2 h after) the colonoscopy.

The tolerability profiles of the bowel preparation agents were assessed. Before the colonoscopy, patients were given a questionnaire (with closed- and open-ended questions) on the (1) taste of their solution (very bad, bad, neutral, good, and very good), (2) their overall satisfaction with the bowel cleansing agent (very comfortable, comfortable, intermediate, uncomfortable, and very uncomfortable), (3) their willingness to use the bowel preparation regimen again (yes and no), and (4) their impression of the bowel preparation agents (open-ended). The rating was on a scale of 1 to 5, ranging from “very good” to “not acceptable,” and the quality of the preparation was rated from “clear stool” to “some fecal materials.” This questionnaire was returned and collected by the staff.

### 2.6. Statistics

The study was designed as a non-inferiority study with a predefined interval of 10% for the percentage of patients with successful bowel preparation. A difference of less than 10% between the efficacy rates of both preparations was considered not clinically relevant.

For the determination of sample size, we assumed that the percentage of patients with a fair preparation would be 80%. A drop-out rate of 10% was anticipated. With a power of 80% and an alpha of 0.05, 220 patients were required in each group.

We analyzed data from the participants who completed colonoscopy. The statistical significance of the differences between groups was calculated using chi-square (nominal distribution) or Fisher’s exact (non-nominal distribution) tests for categorical variables (expressed as number and percentage) and Student’s *t*-tests (nominal distribution) or Mann–Whitney tests for continuous variables (expressed as the mean ± standard deviation). The demographic characteristics, bowel cleansing efficacy (cleansing quality, polyp detection rate, and adenoma detection rate), safety, and tolerability profiles were compared between the groups. A *p* value < 0.05 was considered significant. The analysis of safety and tolerability included all the subjects, except those who were excluded for cancellation or protocol violation.

All the statistical analyses were conducted using SPSS for Windows version 22.0 (IBM Corporation, Armonk, NY, USA).

## 3. Results

### 3.1. Baseline Characteristics

After applying the exclusion criteria and excluding patients who violated the protocol, 440 healthy volunteers were enrolled and randomly assigned to receive either the probiotic solution with bisacodyl (case group, *n* = 220) or 4 L of PEG (control group, *n* = 220). Forty patients dropped out before the screening colonoscopy because they did not properly conduct the bowel preparation protocol (did not follow the follow-up schedule for preparation, consume bisacodyl, properly mix the solution, or ingest more than 80% of the bowel preparation). A total of 385 patients (case, *n* = 195; control, *n* = 190) successfully completed the protocol and underwent colonoscopy.

The demographics, including age, sex, and body mass index, were not significantly different between the groups (Table 1).

### 3.2. Efficacy Profiles of Probiotic Solution with Bisacodyl vs. Conventional 4-L PEG Solution

The overall mean Ottawa score was different between the groups (case: 7.67 ± 2.78 vs. control: 6.42 ± 2.58, *p* = 0.01), but without clinical significance. A high-quality preparation for the screening colonoscopy (Ottawa score <1) was more frequently achieved in the control group (174 patients, 91.58%) than in the case group (168 patients, 86.15%), but not significantly so (*p* = 0.09). When the Ottawa score for each bowel segment was stratified into an adequate vs. inadequate level (Ottawa score ≤3 vs. > 3), there were no differences between the groups in each segment of the colon (right-sided colon: 87.18% vs. 87.37%, *p* = 0.91; descending: 89.74% vs. 95.26%, *p* = 0.43; recto-sigmoid colon: 91.28% vs. 97.37%, *p* = 0.51, in the case vs. control group, respectively) (Table 2).

Ottawa bowel preparation scale: 4 points, inadequate: presence of semisolid or solid stool that leads to incomplete colonoscopy; 3 points, poor: significant amount of semisolid stool was present that could not be removed by rinsing or suction; 2 points, fair: a small amount of semifluid stool was present that could be removed by blow liquid, rinsing, or suction; 1 point, good: some amount of clear fluid removed but good view of the mucosa could be achieved; 0 points, excellent: only a small amount of clear fluid removed in the colon. The amount of liquid in the entire colon: 2 points, high; 1 point, moderate; 0 points, low.

The polyp and adenoma detection rates were not significantly different between groups (38.42% vs. 32.42%, *p* = 0.30; 25.79% vs. 18.97%, *p* = 0.11, respectively) (Table 3).

### 3.3. Safety Profiles of Probiotic Solution with Bisacodyl vs. Conventional 4-L PEG Solution

The case group reported significantly fewer adverse events than the control group, especially nausea, vomiting, and abdominal bloating events (nausea: 32% vs. 70%, *p <* 0.001; vomiting: 6% vs. 20%, *p* = 0.01; abdominal bloating: 50% vs. 72%, *p <* 0.001 in the case vs. control group, respectively).

**Figure 1:**
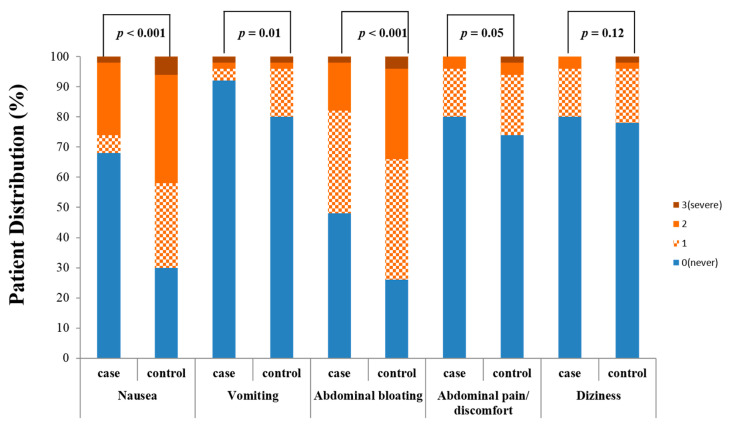
Patient safety profiles for the bowel preparation agents.

The case group also reported fewer abdominal pain/discomfort and dizziness events than the control group, but not significantly so (Figure 1).

### 3.4. Patient Tolerability Profiles of Probiotic Solution with Bisacodyl vs. Conventional 4-L PEG Solution

The case group reported a significant improvement in the taste of the purgative (≥ average; case vs. control: 97% vs. 56%, *p <* 0.001).

**Figure 2:**
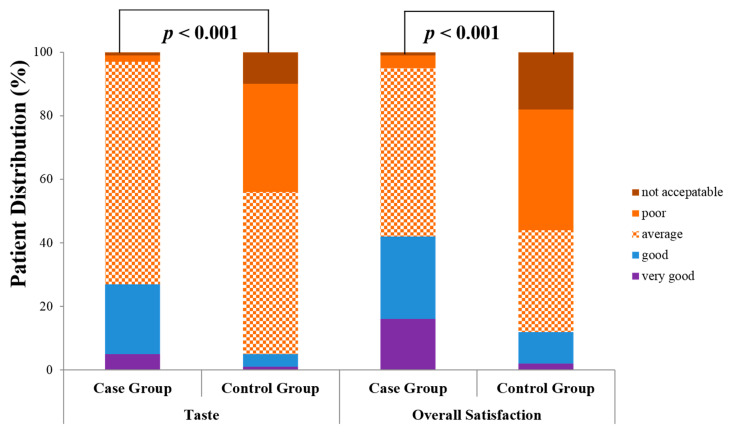
Patient tolerability profiles for bowel preparation agents.

Regarding the overall satisfaction grade for the bowel preparation agents, the case group reported significantly more above “average” scores (≥ average, case vs. control: 95% vs. 44%, *p <* 0.001) (Figure 2).

The case group was significantly more willing than the control group to use the same preparation again (case vs. control: 90.26% vs. 61.85%, *p <* 0.001).

## 4. Discussion

In this prospective, randomized controlled trial, we investigated the efficacy, safety, and tolerability profiles of a probiotic solution with bisacodyl (case group) versus a conventional 4-L PEG solution (control group). To investigate the efficacy profiles, we compared the bowel cleansing quality using the Ottawa scale (inadequate/adequate), polyp detection rate, and adenoma detection rate between groups. Although the proportion of extremely high-quality bowel preparation (Ottawa scale < 1) was predominant in the control group, there was no statistically significant difference between the groups when the bowel preparation scale was stratified into the inadequate and adequate scale, which was generally regarded to meet the minimum condition for screening colonoscopy. The polyp and adenoma detection rates were also not significantly different between groups. Furthermore, in terms of patient satisfaction and tolerability to bowel cleansing agents, the probiotic solution with bisacodyl group showed better patient-reported outcomes than the conventional 4-L PEG solution group. As patient compliance with bowel preparation agents is associated with achieving an adequate level of bowel cleansing, a probiotic solution with bisacodyl might be a new bowel preparation candidate, especially in patients who show a poor compliance with conventional bowel preparation agents.

Patient compliance with bowel cleansing agents is an important step to achieve high-quality bowel preparation; in addition to the efficacy of bowel preparation, patient tolerability for bowel cleansing agents is essential. In this regard, a probiotic solution with a bisacodyl agent might be a candidate for a new bowel preparation, especially for those who do not tolerate conventional bowel preparation agents owing to the taste or large volume of agent required. Additionally, as this new method showed better patient satisfaction during bowel cleansing owing to convenience, those who prefer convenient methods for bowel cleansing may prefer this method.

The possible mechanisms behind the effect of the probiotic mixture solution to reduce the incidence side effects induced by bowel preparation are as follows [38,40,45,46,47]. The probiotic mixture solution might result in improved colon-transit time and further bowel cleansing. Several studies have reported that probiotic mixtures are effective and have beneficial effects for evacuation disorder and constipation and can improve the bowel preparation quality [38,40,45,46]. Probiotics improve dysbiosis and fermentation, increase the production of short-chain fatty acids, and produce gases (H_2_, CO_2_, and CH_4_) [45]. This results in a lower pH in the colonic mucosa, which enhances peristalsis and accelerates the bowel transit time [40,45,46]. In addition to experimental studies, there have been several clinical studies on the effect of probiotics on bowel cleansing [39,48]. For example, Lee et al. investigated the efficacy of a 2-week course of probiotics with oral sodium phosphate in bowel preparation, especially in patients with constipation before colonoscopy, compared with that in the placebo group. The case group (probiotics) showed better bowel cleansing than the placebo group (54.9% vs. 20.8%, *p <* 0.001) [39]. Zia et al. investigated the effects of probiotics in bowel preparation in patients with colorectal cancer for surgery compared with conventional bowel preparation [48]. In this study, they not only evaluated the quality of bowel preparation but also focused on the intestinal barrier on the intestinal epithelium in the colon by microscopic examination and levels of a transmembrane binding protein (occludin) using an immunohistochemistry method [48]. Although the bowel preparation quality of each group was not different, the expression levels of occludin (19.32% ± 2.40% vs. 16.21% ± 2.54%) and IgA (7.60% ± 1.48% vs. 5.29% ± 1.57%) in the colon were significantly higher in the trial group than in the control group [48]. Therefore, the results obtained in our study might be related to fewer complications, including abdominal pain/discomfort, nausea, and vomiting, in the probiotic solution with bisacodyl group than in the control group, and the equal quality of the bowel preparation between groups. Further studies should be guaranteed to reveal the precise mechanisms underlying the role of probiotic mixture solution in bowel cleansing.

In addition to the probiotic mixture solution, the participants in the case group used bisacodyl to maintain the effect of the probiotic mixture solution long enough for the cleansing bowel preparation. The effect of bisacodyl on the bowel preparation quality has been previously investigated. For instance, Adams et al. investigated the effect of bisacodyl on bowel preparation and revealed that bisacodyl significantly reduces the total volume of PEG solution in the conventional method [49]. Clark et al. conducted a meta-analysis of the efficacy of bisacodyl in addition to PEG solution in bowel cleansing and showed that bisacodyl significantly improves the patient compliance and tolerance and the quality of bowel preparation [32]. In our study, a probiotic solution with bisacodyl improved bowel preparation quality compared with the probiotic solution alone in a pilot study (data not shown).

There were several limitations to this study. First, although we conducted this study as a prospective, randomized, controlled trial, as this study was conducted in a single center the composition of patients and staff characteristics affect the generalizability of our results. Further multicenter studies should be conducted. Second, all the enrolled participants were Korean; thus, these results should be applied to other ethnicities with caution. Third, in this study, we enrolled healthy volunteers at the Health Promotion Center who wanted to undergo screening colonoscopy and had no known medical history. Efficacy and safety profiles should be further investigated in patients with severe comorbidities. Fourth, as we performed a screening colonoscopy with one-stage endoscopic mucosal resection or biopsy to remove polyps, the exact endoscopic withdrawal time for each patient was not available. However, in our medical center (Gachon University GMC and Health Promotion Center), approximately 30,000 endoscopies are performed each year and colonoscopy cases account for more than 1/4 of all cases. The GMC Quality Control Unit and Korean Society of Gastroenterology tightly monitor our gastroenterology department to maintain the quality of endoscopy [50,51]. Fifth, in this study, we explored the efficacy of a probiotic mixture solution with bisacodyl in one protocol. As the same bowel preparation solution shows different efficacy profiles with different protocols—such as single dose vs. split dose, different schedules, timings of bowel preparation, and timings of colonoscopy—further investigation to determine the optimal protocol (schedule, dose, and duration) for this new method should be conducted 

There were also strengths to this study. This was the first study to evaluate the efficacy, safety, and patient tolerability profiles of a probiotic solution with bisacodyl compared with a conventional 4-L bowel cleansing agent in a prospective, randomized, case-controlled trial. A probiotic solution (200 mL) with bisacodyl showed better patient-reported outcomes, especially in patient satisfaction with bowel cleansing, because of its convenience. As this new method does not require the ingestion of a large volume of agent but only 200 mL of probiotic mixture solution and a pill of bisacodyl, this simple process for bowel cleansing leads to patient preference. However, it should be noted that there were more extremely high-quality bowel preparation cases in the conventional bowel preparation agent group, although there were no significant differences in the adequacy of bowel preparation between groups, as shown by the Ottawa scale (>3 vs. ≤3), polyp detection rate, and adenoma detection rate. Therefore, this new bowel cleansing method, a probiotic solution with bisacodyl, should be restricted to patients who do not tolerate conventional bowel preparations or show poor compliance with conventional cleansing methods, which lead to barriers to routine periodical screening colonoscopy.

## 5. Conclusions

Our study showed the possibility of using a probiotic solution with bisacodyl as a new bowel preparation method, especially for patients who do not tolerate conventional bowel cleansing agents. As the presence of a variety of choices for bowel preparation agents that meet the preferences of different patients might lead to better compliance for screening colonoscopy, this method might help patients who do not tolerate or comply with conventional bowel preparation before screening colonoscopy. Further studies should be conducted and combination solutions developed to support our results.

## Figures and Tables

**Table 1:** Patient characteristics.

	Case Group (Bisacodyl + Probiotic Solution Group, *n* = 195)	Control Group (PEG Solution Group, *n* = 190)	*p* Value
Age (year)	47.94 ± 9.86	49.03 ± 9.86	0.28
Height (cm)	166.05 ± 8.63	165.78 ± 8.25	0.75
Weight (kg)	67.32 ± 12.01	66.80 ± 11.87	0.67
BMI (kg/m^2^)	24.30 ± 3.08	24.17 ± 2.99	0.68

Abbreviation: PEG, polyethylene glycol; BMI, body mass index.

**Table 2:** Comparisons of the efficacies of bowel preparation agents according to the bowel segments between groups using the Ottawa bowel preparation scale¶.

	Ottawa Scale Score	Case Group (Bisacodyl with Probiotics Group, *n* = 195)	Control Group (PEG Group, *n* = 190)	*p* Value
Total Ottawa score		7.67 ± 2.78	6.42 ± 2.58	0.01
Right sided colon, *n* (%)	Adequate	170 (87.18%)	166 (87.37%)	0.91
	poor	25 (12.82%)	24 (12.63%)	
Descending colon, *n* (%)	Adequate	175 (89.74%)	181 (95.26%)	0.43
	poor	20 (10.26%)	9 (4.74%)	
Recto-sigmoid colon, *n* (%)	Adequate	178 (91.28%)	185 (97.37%)	0.51
	poor	17 (8.72%)	5 (2.63%)	
Fluid, *n* (%)	Adequate	144 (73.68%)	121 (63.59%)	0.36
	poor	51 (26.32%)	69 (36.41%)	

Abbreviation: PEG, polyethylene glycol.

**Table 3:** Comparisons of the efficacies in the polyp detection rate according to the bowel segments between groups.

	Case Group (Probiotics Group, *n* = 195)	Control Group (PEG Group, *n* = 190)	*p* Value
Polyp detection rate, *n* (%)	32.82%	38.42%	0.30
Adenoma detection rate, *n* (%)	18.97%	25.79%	0.11

Abbreviation: PEG, polyethylene glycol.

## Data Sharing and Data Accessibility

The data used to support the findings of this study are available from the corresponding author upon request.
